# Temporopolar volumes are associated with the severity of social impairment and language development in children with autism spectrum disorder with developmental delay

**DOI:** 10.3389/fpsyt.2022.1072272

**Published:** 2022-12-01

**Authors:** Yiting Ji, Mingyu Xu, Xin Liu, Yuan Dai, Li Zhou, Fei Li, Lingli Zhang

**Affiliations:** ^1^Department of Child and Adolescent Healthcare, Children’s Hospital of Soochow University, Suzhou, Jiangsu, China; ^2^Department of Developmental and Behavioral Pediatric & Child Primary Care, Brain and Behavioral Research Unit of Shanghai Institute for Pediatric Research, MOE-Shanghai Key Laboratory for Children’s Environmental Health, Xinhua Hospital, Shanghai Jiao Tong University School of Medicine, Shanghai, China; ^3^Psychology and Neuroscience of Cognition Research Unit, University of Liège, Liège, Belgium

**Keywords:** autism spectrum disorder, developmental delay, gesture, joint attention, temporal pole

## Abstract

**Background:**

Children with autism spectrum disorder (ASD) and developmental delay (DD; ASD + DD) have more severe clinical symptoms than those with ASD without DD (ASD-only). However, little is known about the underlying neuroimaging mechanisms. The aim of this study was to explore the volumetric difference between patients with ASD + DD and ASD-only and investigate the relationship between brain alterations and clinical manifestations.

**Materials and methods:**

A total of 184 children with ASD aged 2–6 years were included in this study, who were divided into two groups according to their cognitive development: ASD + DD and ASD-only. Clinical symptoms and language development were assessed using the Autism Diagnostic Observation Schedule (ADOS), Childhood Autism Rating Scale (CARS), and the Putonghua Communicative Development Inventory. Of the 184 children, 60 age-matched males (30 ASD + DD and 30 ASD-only patients) with high-resolution structural neuroimaging scans were included for further voxel-based morphometry analysis to examine the relationship between clinical symptoms and gray matter volumes.

**Results:**

The ASD + DD group had higher CARS and ADOS scores, lower gesture scores, and poorer performance in “responding to joint attention” (RJA) and “initiating joint attention” than the ASD-only group. Larger gray matter volumes in the temporal poles of the right and left middle temporal gyri were associated with the co-occurrence of DD in patients with ASD. Moreover, temporopolar volumes were correlated with CARS and ADOS scores, gesture scores, and RJA ability. Pre-language development significantly mediated the relationship between temporopolar volumes and both CARS and ADOS scores; RJA ability, but not gesture development, contributed to this mediating effect.

**Conclusion:**

In this study, we found that temporopolar volumes were enlarged in patients with ASD who had comorbid DD, and these patients showed an association between symptom severity and language ability during the pre-language stage. Offering early interventions focused on RJA and the temporal pole may help improve clinical symptoms.

## Introduction

Autism spectrum disorder (ASD) is a complex neurodevelopmental disorder characterized by social communication deficits, repetitive behaviors, and restricted interests. The previous several decades have seen a significant increase in the prevalence of ASD. The most recent report in 2021 suggested that the prevalence of autism had reached 1/44 in the United States (1). Currently, ASD is one of the most common neurodevelopmental disorders, affecting approximately 78 million people and their families worldwide. ASD is a highly heterogeneous condition with differential manifestations of language development, intelligence, and comorbidity, which influence intervention plans. A recently published Lancet commission proposed the concept of “profound autism”, referring to autism with severe to profound intellectual disability (ID) or minimal language, for which additional support is required because of the patient’s inability to live independently (2). The commission suggested that more studies focused on autism with ID and the underlying mechanisms are needed, which would be more valuable for improving clinical and societal outcomes.

To study the early clinical manifestation of profound autism and understand the behavioral heterogeneity of ASD, there is no better example than variability in early language development (3). For toddlers and pre-schoolers, delays in language development are one of the primary concerns that motivate parents to seek ASD diagnostic evaluation for their children, especially those comorbid with DD/ID (3, 4). Moreover, deficits in early language developmental trajectories, such as the use of gestures and the ability to engage in joint attention, have been observed in children with ASD (5, 6). Gestures are a form of pre-verbal communication through body movements (such as pointing and showing), providing a foundation upon which communication skills can further develop; while joint attention involves sharing attention with a partner to a third entity by integrating gestures and eye gaze, which include the ability to follow others’ attention (response to joint attention, RJA) and initiating joint attention (IJA) (7–9). Therefore, pre-language developmental discrepancy may be an important contributor to phenotypic heterogeneity between young ASD with and without DD/ID (10), and the assessment of gesture and joint attention in this critical period is crucial either to understanding their early communicative skills and for planning prompt intervention when the brains are still in development (11–13).

Neurobiological explanations for why toddlers and pre-schoolers with ASD exhibit striking differences between those with and without comorbid DD/ID are lacking. Non-invasive imaging techniques are promising tools to investigate the neurological underpinnings of ASD; and these neuroimaging characters may be served as potential biomarkers to discriminate patient subgroups and intervention targets. In patients with autism aged 2–65 years, cortical thickness across the frontal, temporal, and occipital cortices has been shown to be negatively associated with the full intelligence quotient score and overall clinical symptom severity (14). Moreover, a functional connectivity study showed lower network segmentation and integration in patients with ASD whose ages ranged from 7 to 17 with low verbal and cognitive performance than in those with high verbal and cognitive performance (15). The current pieces of evidence from ASD patients with a broad age range indicate potential neuroimaging characteristics of ASD with differential cognitive function; however, considering the high plasticity of the brain in development, such neuroimaging evidence on young children with ASD and the relationship between brain alterations and impaired early language development remains scarce.

In the present study, we aimed to compare the features of early-language development profiles between the two subgroups of ASD with DD (ASD + DD) and ASD-only and identify brain volume differences between these two groups. We also examined the relationship between behavioral heterogeneity and brain structural alterations. It provides an important complement to previous related research. This study serves as an important step toward parsing factors that influence neuroanatomical heterogeneity in ASD and is a potential step toward establishing precise medical biomarkers.

## Materials and methods

### Participants

Autism spectrum disorder participants were enrolled in the Shanghai Xinhua ASD Registry in Shanghai, China. Participants were recruited from January 2016 to December 2017. Children aged 2–6 years were diagnosed with ASD according to the *Diagnostic and Statistical Manual of Mental Disorders*, Fifth Edition (DSM-V) (16). Diagnoses were confirmed with the Autism Diagnostic Observation Schedule (ADOS), the Autism Diagnostic Interview-Revised (ADI-R), and a Childhood Autism Rating Scale (CARS) total score of no less than 30. In addition, children were excluded if they met any of the following criteria: chromosomal or genetic abnormalities; hearing or visual impairments; diagnosed with neurological disease (e.g., epilepsy and Rett syndrome); and diagnostic imaging anomalies if the patient had undergone magnetic resonance imaging (MRI) scanning.

A total of 184 children were enrolled in the study. Children were classified as ASD with DD (ASD + DD) or ASD-only according to whether they presented with DD/ID (developmental quotient [DQ] and/or IQ score < 75). Since the sex/gender differences in ASD have been observed in previous neuroimaging studies (17, 18), and the sample size of the ASD female participants who had MRI scans was limited in our study, we focused on the ASD men for further neuroimaging analysis; 60 age-matched male participants (30 ASD + DD and 30 patients with ASD-only) scanned with the Siemens Version 3.0-Tesla MRI scanner were included.

The parents or guardians of all participants provided written informed consent according to the Declaration of Helsinki. This study was approved by the Ethical Committee of Shanghai Jiao Tong University School of Medicine, affiliated with Xinhua Hospital.

### Behavioral assessments

#### Autistic symptoms

Autism Diagnostic Observation Schedule and CARS were used for the diagnosis of ASD alongside the DSM-V and ADI-R by a qualified and experienced assessor. These scales were also used to evaluate the clinical severity of autistic symptoms, where a higher ADOS score and CARS total score indicated more severe symptoms. For ADOS, a calibrated ASD severity score (CSS) was also calculated in the social affect (SA) domain to represent the severity of social communication.

#### Cognitive function

The average DQ in children aged 2–4 years was evaluated using the Gesell Developmental Diagnosis Scale (GDDS), whereas the IQ of children aged 4–6 years was assessed using the Weschler pre-school and primary scale of intelligence (19). We have compared the language development between the two groups using the language DQ in GDDS for children under 4 years.

#### Gesture development

The Putonghua Communicative Development Inventory (PCDI), adapted for Mandarin Chinese, was used to assess the early language and communication development of children aged 8–30 months, and it can be used to assess older children with developmental disorders (20, 21). The form of “Words and Gestures” was used to assess children’s early communication ability according to their actual mental age and language ability. “Words and Gestures” was divided into two sections, of which the second section was used to evaluate the use of gestures. This section included three items: early gestures, late gestures, and total gestures. Gestures on the PCDI that appear early reflect social communication, whereas gestures that appear later reflect symbolic ones (6). A higher score indicated a better level of gesture development.

#### Joint attention

“Responding to joint attention” (RJA) and “initiating joint attention” (IJA) were evaluated within the ADOS assessment. For the scenario of RJA, the evaluator assessed the child’s response to the use of gaze coordinated with facial orientation, vocalization, and pointing to draw attention to a distant object. When rating the IJA item, the evaluator assessed whether the child used eye-tracking or oral language to draw adults’ attention. Scores (0, 1, 2, or 3) represented different degrees of engagement (e.g., coordinated joint engagement with gaze shift, joint engagement with gaze shift and pointing, and unengaged) and initiation (e.g., child-initiated) (22).

### Image acquisition and pre-processing

#### Image acquisition

Participants were scanned using a Siemens Version 3.0-Tesla MRI scanner (Siemens Medical Solutions, Munich, Germany) with a 32-channel head coil and a four-channel neck coil. Given their young age and inability to cooperate during the examination, our subjects were under sedation (50 mg/kg chloral hydrate at a maximum dose of 1 g administered rectally). Additionally, earplugs, earphones, and extra foam padding were provided to the participants to reduce the impact of the sound of the scanner during the scan. Through these efforts, the images of all patients were clear and usable. The high-resolution anatomical T1-weighted magnetization-prepared rapid gradient echo image (192 sagittal slices; voxels = 1 × 1 × 1 mm; repetition time [TR] = 2,300 ms; echo time [TE] = 2.28 ms; inversion time = 1,100 ms; flip angle = 8°; and field of view = 192 × 192 × 192 mm) was acquired.

#### Image pre-processing

T1-weighted images were processed using SPM12^1^ and the CAT12 toolbox, which incorporates the DARTEL toolbox. Pre-processing steps included segmentation, registration, normalization, and smoothing. Notably, customized tissue probability maps (TPMs) of children were created using the Template-O-Matic Toolbox.^2^ These customized TPMs were used for the initial spatial registration and segmentation processes. We used the standard optimized method for iterative tissue segmentation and spatial normalization, which uses both linear (12-parameter affine) and non-linear transformations. To ensure that the residuals in later analyses conform to a Gaussian distribution and to account for individual differences in brain anatomy, the modulated gray matter images in Montreal Neurological Institute (MNI) space were smoothed using an isotropic Gaussian kernel of 8 mm full width at half maximum. The resulting voxel size was 1.5 × 1.5 × 1.5 mm^3^.

### Statistical analyses

#### Analysis of clinical symptoms

The SPSS version 21.0 statistical software (Chicago, IL, United States) was used for clinical data analysis. After testing for data distribution and variance homogeneity, we calculated the means ± standard deviations for all measurement data, which included age, the GDDS average DQ, the ADOS social + communication score, the CARS total score, and the scores of the PCDI. Comparisons between the two groups were conducted using independent-sample *t*-tests. Classification data, such as sex, RJA, and IJA item scores, are expressed as numbers and were compared using χ^2^ tests. A *p*-value <0.05 was considered significant.

#### Analysis of brain volumes

##### Structure association analysis

A linear regression model was used to investigate the relationship between gray matter volume (GMV) and ASD with/without DD, with age and total intracranial volume as covariates of no interest. For the neuroimaging analysis, we conducted a permutation-based cluster-level correction (5,000 times) for multiple comparisons (23). At the voxel level, we used a two-sided test with a significance level of α = 0.001, whereas at the cluster level, we used a permutation-based family-wise error correction with a significance level of α = 0.05. Significant GMVs were defined as clusters with more than 217 voxels that fell within the 90% confidence interval (CI) of the smoothing kernel voxels (24, 25). An association analysis was then conducted between significant GMVs and clinical symptoms of autism and language development scores.

##### Mediation analysis

We tested the mediating effect of language development scores on the association between significant GMVs and clinical symptoms of autism while controlling for age and total intracranial volume as covariates of no interest. We applied a bootstrap procedure provided by PROCESS in SPSS^3^ to test the mediation (indirect) effect. The indirect effect was considered significant if the bootstrap CI did not include zero.

## Results

### Demographic and clinical characteristics of the participants

A total of 184 participants were enrolled from the Shanghai Xinhua Registry, which consisted of 103 patients with ASD + DD and 81 patients with ASD-only. There was no significant difference in age (*t* = −0.045, *p* = 0.964) or sex (χ*^2^* = −0.990, *p* = 0.569) between the two groups. The CARS total score, ADOS social + communication score differed significantly between the ASD + DD and ASD-only groups (CARS: *t* = 7.029, *p* < 0.001; ADOS: *t* = −2.548, *p* = 0.012). The ASD + DD group (CARS: 38.81 ± 3.86; ADOS: 17.16 ± 3.59) had higher CARS, and ADOS scores than the ASD-only group (CARS: 34.60 ± 4.17; ADOS: 15.86 ± 3.17). In addition, compared with ASD-only, higher ADOS-SA CSS were observed in patients with ASD + DD (*t* = −2.207, *p* = 0.029) (Table 1). The language DQ in GDDS between the two groups was different (*t* = 12.988, *p* < 0.001).

**TABLE 1:** Comparisons of demographic characteristics and symptom severity in autism spectrum disorder with or without developmental delay.

Group Mean (SD)	ASD + DD (*N* = 103)	ASD-only (*N* = 81)	χ*^2^/t*	*P*-value
Age, months	36.35 (7.82)	36.41 (9.13)	–0.045	0.964
Sex			0.990	0.569
Male *n* (percentage)	84 (81.6%)	66 (81.5%)		
GDDS Average DQ	57.69 (10.38)	83.71 (7.40)	–19.290	< 0.001**
Language DQ in GDDS	71.29 (17.67)	40.52 (11.99)	12.988	< 0.001**
CARS total score	38.81 (3.86)	34.6 (4.17)	7.029	< 0.001**
ADOS social + communication score	17.16 (3.59)	15.86 (3.17)	–2.548	0.012*
ADOS-SA CSS	8.84 (4.17)	8.31 (1.75)	–2.207	0.029*

SD, standard deviation; ASD, autism spectrum disorder; DD, development delay; GDDS, Gesell Developmental Diagnosis Scale; DQ, developmental quotient; ADOS, Autism Diagnostic Observation Schedule; CSS, calibrated ASD severity score; SA, social affect. Group comparison is significant at the 0.05 level.

**P<0.01; *P<0.05.

### Comparisons of gesture development between autism spectrum disorder patients with and without developmental delay

The early, later, and total gesture scores were significantly different between the two groups (*p* < 0.05). Compared with the ASD-only group, the ASD + DD group’s scores for early, later, and total gestures were lower (Table 2).

**TABLE 2:** Comparisons of gesture development between autism spectrum disorder patients with and without gesell developmental diagnosis scale.

Mean (SD)	ASD + DD (*N* = 103)	ASD-only (*N* = 81)	*t*	*P*-value
Early gesture score	13.02 (6.76)	15.13 (6.00)	–2.000	0.047*
Later gesture score	16.78 (6.06)	19.42 (5.56)	–2.742	0.007**
Total gesture score	29.63 (11.60)	34.27 (10.95)	–2.496	0.014*

SD, standard deviation; ASD, autism spectrum disorder; DD, development delay. Group comparison significant at the 0.05 level.

**p<0.01; *p<0.05.

### Comparisons of responding to joint attention and initiating joint attention ability between autism spectrum disorder patients with and without developmental delay

The comparisons of RJA and IJA ability showed that the ASD + DD group was poorer than the ASD-only group (*Z* = 27.630, *p* < 0.001; *Z* = 15.981, *p* < 0.001). For the RJA scores, 3.9% of patients in the ASD + DD group and 17.3% of patients in the ASD-only group had a score of zero.

**FIGURE 1:**
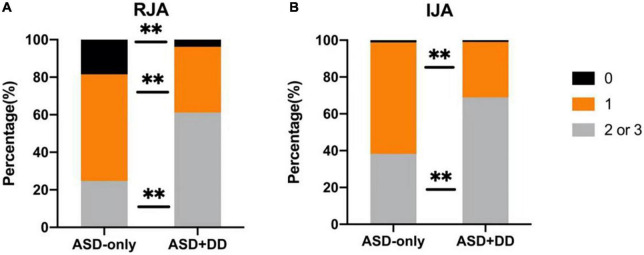
Comparisons of responding to joint attention (RJA) and initiating joint attention (IJA) ability between autism spectrum disorder (ASD) patients with and without developmental delay (DD). **(A)** and **(B)** represent the percentages of RJA and IJA scores. Comparisons between the two groups were performed using an analysis of variance. Different colors represent different scores. Black indicates an alerting score of 0, orange a score of 1, and gray a score of 2 or 3. **p < 0.01.

However, 62.1 and 25.9% of the ASD + DD and ASD-only patients, respectively (Figure 1A), had higher RJA scores (i.e., two or three), which indicate more severe symptoms, and 68.9 and 38.3% of the patients with ASD + DD and ASD-only, respectively, had higher IJA scores (Figure 1B).

### A larger temporopolar cortex is associated with the co-occurrence of developmental delay in patients with autism spectrum disorder

**FIGURE 2:**
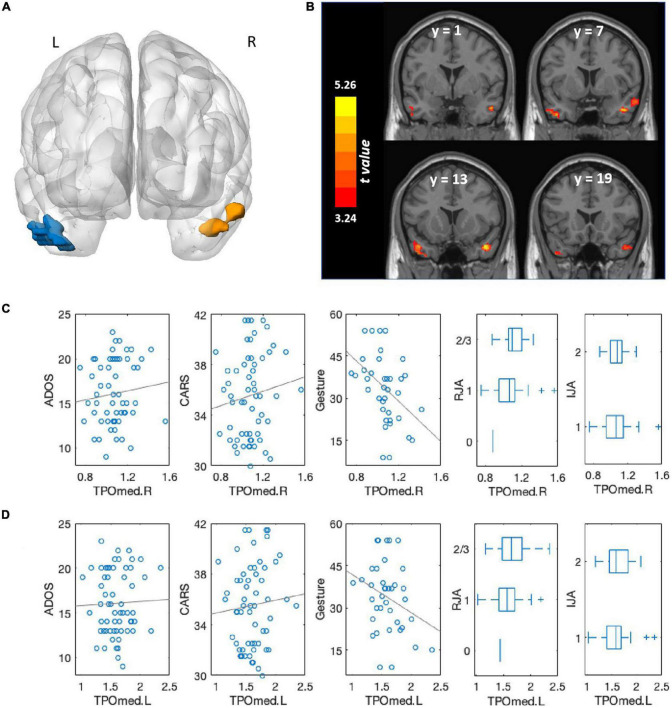
Comparison of brain volumes between autism spectrum disorder (ASD) patients with and without developmental delay (DD) and associations with clinical symptoms. **(A)** Brain regions that significantly differed between ASD patients with and without DD. The blue region comprised the TPOmed.L and left inferior temporal gyri; the orange region primarily comprised the TPOmed.R. Multiple comparison corrections were *p* < 0.001 at the voxel level and *p* < 0.05 cluster-level family-wise error correction. **(B)** Brain regions that significantly differed between ASD patients with and without DD, shown in the coronal view. The color bar represents *t*-values. **(C)** Association between TPOmed.R volume and ADOS scores, CARS scores, gestures scores, RJA ability, and IJA ability across the whole group. **(D)** Association between TPOmed.L volume and ADOS scores, CARS scores, gestures scores, RJA ability, and IJA ability across the whole group.

Volumetric differences were observed between the ASD + DD and ASD-only groups in two brain regions: mainly distributed across the temporal poles of the right middle temporal gyrus (TPOmed.R) and the left middle temporal gyrus (TPOmed.L) (Figure 2 and Table 3).

**TABLE 3:** Comparison of gray matter volume (GMV) between autism spectrum disorder (ASD) patients with and without developmental delay (DD).

Region	Cluster size (voxels)	Peak *t* value	Peak MNI		
			X	Y	Z
Temporal_Pole_Mid_L(TPOmed.L)/Temporal_Inf_L	744	4.56	–43.4	7.5	–37.5
Temporal_Pole_Mid_R (TPOmed.R)	608	5.36	43.5	12	–30

Multiple comparison correction was *p* < 0.001 at the voxel level and *p* < 0.05 cluster-level family-wise error correction. Temporal_Pole_Mid_L, the temporal poles of the left middle temporal gyrus; Temporal_Inf_L, left inferior temporal gyrus; Temporal_Pole_Mid_R, the temporal poles of the right middle temporal gyrus.

For the association between the volumes of these two regions and clinical symptoms, we found that a larger TPOmed.R volume was correlated with greater severity of autistic symptoms as measured by both the ADOS score (*r* = 0.343, *p* = 0.008) and the CARS total score (*r* = 0.303, *p* = 0.021). A marginally significant association was observed between TPOmed.L volume and clinical symptoms (ADOS: *r* = 0.251, *p* = 0.057; CARS: *r* = 0.231, *p* = 0.082).

We also found that a larger TPOmed volume was correlated with a poorer gesture score (right: *r* = −0.624, *p* < 0.001; left: *r* = −0.443, *p* = 0.007) and RJA ability (right: *r* = 0.352, *p* = 0.007; left: *r* = 0.283, *p* = 0.031), whereas no correlation was observed between TPOmed volume and IJA ability (right: *r* = 0.140, *p* = 0.303; left: r = 0.248, *p* = 0.065).

### Relationship among temporopolar volume, language development, and autistic symptoms

**FIGURE 3:**
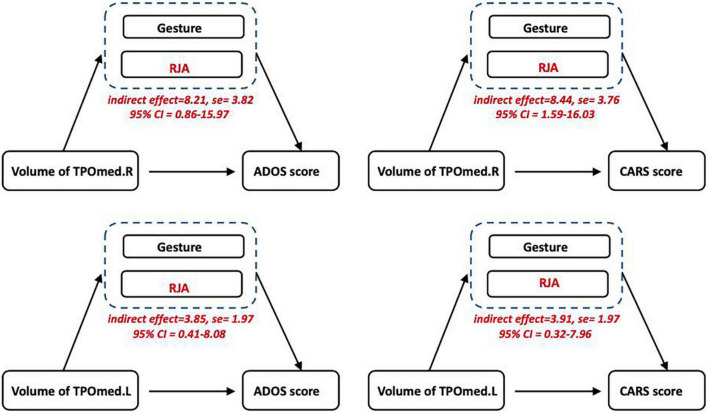
Relationship among temporopolar volume, language development, and autistic symptoms. The mediation analysis showed an association between the TPOmed volume and ASD severity (CARS and ADOS scores), which was mediated by RJA ability, but not gesture development.

The mediation analysis was employed to examine whether language development mediated the relationship between TPOmed volume and autistic clinical symptoms. We observed a significant mediating effect of pre-language development on the association between TPOmed.R volume and both ADOS and CARS scores. In addition, we found that RJA ability, but not gesture development, contributed to this mediation effect. Similarly, the ability of RJA also linked TPOmed.L volume to poor clinical performance (Figure 3).

## Discussion

In this study, we noted that ASD children comorbid with DD/ID exhibited more severe clinical symptoms and more profound delay in gesture and joint attention than those without DD/ID, which indicated the importance of early surveillance of pre-language development. The volumetric alterations in the temporal poles of the middle temporal gyri worked as a driving neuroimaging factor for poor RJA ability and more severe clinical symptoms, indicating a potential brain intervention target to improve the clinical manifestations of ASD with DD/ID.

Currently, within the context of the novel concept of profound autism, increased attention has been drawn toward ASD children with comorbid DD/ID, since most of them are unable to live independently; the life-long cost of profound autism in education and healthcare was a heavy burden to both the families and the society (26). Although this concept is not appropriate to be applied to younger children, given their high plasticity of the brain and behavior, early recognition and intervention for disruptions in the developmental milestones associated with DD/ID in children with autism are necessary, which may improve the developmental trajectory pathways and prognosis of profound autism. We found that children with ASD + DD/ID showed more severe clinical symptoms compared with ASD-only (27), which manifested in the early developmental stage. Additionally, we focused on pre-verbal social communication skills and found widespread impairments in gesture, RJA, and IJA in children with ASD with comorbid DD/ID (5, 28). In typical development, the production of deictic gestures emerges early, around 8–10 months, and the engagement of joint attention can appear in the first year of life. Studies found that infants aged 15–18 months who were later diagnosed with autism used fewer gestures and joint attention than infants who were found to have DD and typical development (8). Therefore, deviation in early language development was pervasive in ASD + DD/ID and early language ability could be an important clinical feature and prognostic predictor of ASD with comorbid DD/ID (26).

Notably, the pre-language developmental discrepancy may be an important contributor to phenotypic heterogeneity between young ASD with and without DD/ID, and the assessment of gesture and joint attention in this critical period is crucial either in understanding their early communicative skills or developing suitable intervention when the brains are still in development. Early interventions targeting joint attention can help improve language and social skills in ASD with limited language (29). Some promising interventions, such as Joint Attention, Symbolic Play, Engagement and Regulation (known as JASPER), and the Pre-school Autism Communication Trial (known as PACT), which are interventions targeted at joint attention and parent-children synchrony, have been shown to alleviate both the core symptoms and clinical severity of ASD, and these effects can be maintained over time (30, 31). It is well-accepted that JASPER intervention could improve joint attention, joint engagement, and play directly in children with ASD (31). Another intervention, PACT, was also focused on joint attention and could result in long-term alleviation both in social communication and repetitive symptom domains for young children with ASD (30). These findings indicate that pre-language functions, including gesture and joint attention, and the related brain region may serve as a potential prognostic and therapeutic target, especially for ASD patients with comorbid DD/ID.

In our study, we also found that the differential brain region identified between ASD children with and without DD/ID was the temporal pole, and the ability of RJA mediated the relationship between temporopolar volume and symptom severity. This observation is in line with previous studies. The temporal pole is a cortex capable of higher-order cognitive functions and mentalizing processes, and the engagement of the temporal pole in joint attention has been observed in previous neuroimaging studies (32, 33). Previous studies revealed that temporal activation was associated with RJA ability (34). RJA and related behaviors appear to be closely associated with parietal and temporal cortical processes, which can regulate attention orienting to perceived objects or events (35). Furthermore, using a functional MRI eye-tracking paradigm, abnormal activation of the temporal pole was observed during a joint attention task (36). Compared with toddlers with ASD who had normal language development, those with poor language outcomes had hypoactive superior temporal cortices (37). Moreover, Meresse et al. found that cerebral perfusion in the temporal lobe of children with ASD was significantly negatively correlated with ADI-R scores, where the lower the cerebral perfusion in the temporal lobe, the more severe the autism symptoms (38). These present findings may have clinical implications that the temporal pole may serve as a potential prognostic and therapeutic target, especially for ASD patients with comorbid DD. In recent years, non-invasive neuromodulation techniques such as transcranial magnetic stimulation (TMS) have attracted particular interest in treating ASD, and existing evidence support that, *via* modulating certain brain areas or circuits, TMS could be a safe therapeutic option to treat the core symptoms of ASD (39). For example, repetitive TMS to the dorsomedial prefrontal cortex yielded a reduction in social impairment and social anxiety in patients with ASD (40). Therefore, our results indicate that for autistic children comorbid with DD, the temporal pole could be a candidate brain target for intervention and may produce a positive effect on joint attention.

## Limitations

Our research has several limitations. First, the study design was cross-sectional, which limited the exploration of the trajectories of pre-language development in autistic children with language delays. Second, since the neuroimaging analysis was only performed on ASD male participants, the neuroimaging findings in this study could only be extrapolated to the male population. Finally, RJA and IJA abilities were recorded and coded when performing ADOS; combining tools, such as Short Play and Communication Evaluation (SPACE) or eye-tracking (5, 41), would be more informative in exploring joint attention in ASD.

## Conclusion

Temporopolar volumes were significantly enlarged in children with minimal language children and ASD + DD, and were related to ASD severity and language ability during the pre-language stage. Our findings suggest that volumetric differences in the temporal pole may provide a biological basis for autism severity before the onset of clinical symptoms and offer clues for developing interventions targeting the temporal pole, which may improve the prognosis of ASD.

## Data availability statement

The data of this study are available under reasonable and ethically approved request to the corresponding authors.

## Ethics statement

This study involving human participants was approved by the Ethical Committee of Shanghai Jiao Tong University School of Medicine Affiliated Xinhua Hospital. The parents or guardians of all participants provided written informed consent according to the Declaration of Helsinki in the study.

## Author contributions

LZa, FL, YJ, and MX designed the study. YJ, XL, YD, and LZo collected the clinical information and brain imaging data. YJ and LZa carried out the analysis. YJ, MX, and LZa interpreted the results and wrote the manuscript. LZa and FL guided and supervised all work. YJ, MX, XL, YD, LZo, FL, and LZa read, provided the feedback, discussed and approved the final manuscript.

## Footnotes

1
http://www.fil.ion.ucl.ac.uk/spm/

2
https://neuro-jena.github.io/software.html#tom

3
https://www.processmacro.org/index.html
